# Old Versus New: A Historical Comparison of Anaesthesia Trends for Total Knee Arthroplasty and Patient Outcomes

**DOI:** 10.1155/prm/2115652

**Published:** 2026-06-28

**Authors:** Brigid Brown, Maxim Nagorny, Joseph Jang, Emily H. Jaarsma, Tim Soon Cheok, Charlotte Hall, Christopher Horwood, Hidde M. Kroon, Ruurd L. Jaarsma, D.-Yin Lin

**Affiliations:** ^1^ Department of Anaesthesia and Pain Medicine, Flinders Medical Centre, Adelaide, South Australia, Australia, flinders.sa.gov.au; ^2^ College of Medicine and Public Health, Flinders University, Adelaide, South Australia, Australia, flinders.edu.au; ^3^ Department of Surgery and Perioperative Care, University of Texas at Austin, Austin, USA, utexas.edu; ^4^ Department of Orthopaedic and Trauma Surgery, Flinders Medical Centre, Adelaide, South Australia, Australia, flinders.sa.gov.au; ^5^ Department of Clinical Epidemiology, Flinders Medical Centre, Adelaide, South Australia, Australia, flinders.sa.gov.au; ^6^ Department of Surgery, Royal Adelaide Hospital, Adelaide, South Australia, Australia, rah.sa.gov.au; ^7^ Faculty of Health and Medical Sciences, University of Adelaide, Adelaide, South Australia, Australia, adelaide.edu.au

**Keywords:** anaesthesia, pain management, postoperative care, regional, retrospective studies, total knee arthroplasty

## Abstract

**Purpose:**

Total knee arthroplasty (TKA) is a widely performed procedure for end‐stage knee osteoarthritis, with anaesthesia techniques evolving significantly in recent decades. This retrospective study examines trends in anaesthetic practice and postoperative outcomes at an Australian tertiary hospital network, using Acute Pain Service (APS) data from two distinct time periods (1999–2004 and 2018–2022).

**Methods:**

Elective TKA patients enrolled in the acute pain service database from January 1999 to December 2004 (Cohort 1) and January 2018 to December 2022 (Cohort 2) were included. Baseline demographics and anaesthetic practices were compared descriptively. Postoperative outcomes were analysed using mixed‐effects regression models adjusted for demographic and perioperative factors, with hospital included as a random effect. Outcomes included invasive pain management strategies, opioid consumption, pain scores, time to mobilisation, APS review duration and length of hospital stay.

**Results:**

A total of 1273 patients were included (150 in Cohort 1 and 1123 in Cohort 2). Use of spinal anaesthesia increased significantly in Cohort 2 compared with Cohort 1 (96.4% versus 24.0%, *p* < 0.001), as did the adoption of regional anaesthesia techniques (74.3% versus 1.3%, *p* < 0.001). After adjustment, patients in Cohort 2 had significantly lower odds of requiring invasive pain management strategies (OR: 0.02 and 95% CI: 0.00–0.09; *p* < 0.001), mobilised earlier (*β*: −0.39 days, *p* = 0.009), required fewer days of APS review (*β*: −0.59 days, *p* < 0.001) and had shorter hospital stays (*β*: −2.44 days, *p* = 0.001). However, postoperative day one opioid consumption was higher in Cohort 2 (*β*: 55.19 mg oral morphine equivalents, *p* < 0.001), and movement‐related pain scores were modestly increased (*β*: 1.00, *p* = 0.02). Resting pain scores and opioid‐related side effects were comparable between cohorts.

**Conclusions:**

Over two decades, anaesthetic practice for TKA has shifted substantially toward neuraxial and motor‐sparing regional techniques. These changes were independently associated with earlier mobilisation, reduced reliance on invasive pain strategies and shorter hospitalisation, despite higher early postoperative opioid use. The findings reflect evolving international practice patterns and highlight the importance of ongoing evaluation of perioperative care pathways.

## 1. Introduction

Total knee arthroplasty (TKA) is a widely performed surgical procedure for the management of advanced osteoarthritis, offering significant improvements in pain, function and overall quality of life [1]. In Australia, the annual number of TKA procedures has steadily increased. National modelling based on Australian Orthopaedic Association National Joint Replacement Registry data from 2013, combined with population projections, estimates a 276% increase in primary knee arthroplasty volume by 2030, rising from 42,920 to 161,231 procedures [2]. TKA is often undertaken in patients of an increased age with multiple medical comorbidities and carries a perioperative mortality rate of approximately 0.4% [3]. Over the past 2 decades, considerable efforts have been made to enhance patient safety and surgical outcomes. Innovations in surgical technique, reductions in operative time, reduced transfusion requirements and protocols encouraging earlier mobilisation have collectively contributed to a decline in postoperative morbidity and mortality [4].

Parallel to these surgical advances, anaesthetic practices have also evolved. There has been a notable shift toward the use of neuraxial and regional anaesthesia to optimise postoperative analgesia, minimise opioid consumption and facilitate earlier rehabilitation [5]. These developments align with enhanced recovery after surgery (ERAS) protocols and a broader focus on multimodal pain management strategies in the field of anaesthesia [6].

This study presents a retrospective comparative analysis of an Acute Pain Service (APS) database from a major Australian tertiary centre operating across two locations. The primary objective was to examine trends in anaesthetic practice for elective TKA between two distinct time periods: 1999–2004 and 2018–2022, in order to quantify changes in anaesthetic practice and analgesic approaches over the past two decades. By analysing shifts in anaesthetic techniques and their association with key postoperative outcomes, including pain scores, opioid use, mobilisation time and length of stay in hospital, the research aims to highlight the impact of evolving perioperative strategies on patient safety and recovery.

## 2. Methods

This multicentre retrospective analysis was conducted at two large tertiary teaching hospitals—Flinders Medical Cenre and Noarlunga Hospial—located in Adelaide, Australia. The APS was established in 1999 under the Department of Anaesthesia and Pain Medicine to provide perioperative analgesic support. During the first decade of this service, referral criteria were based on clinician discretion and defined as “any patient who a clinician thought could benefit from APS input.” Though referral criteria were not formalised at this time, anecdotal reports from senior anaesthetists suggest that the majority of the patients undergoing TKA were referred to APS during this period. From 2009 onwards, referral was mandatory for all patients receiving spinal anaesthesia, regional nerve blocks or undergoing procedures associated with a high risk of significant postoperative pain, including all TKAs. A consistent team of anaesthetists and orthopaedic surgeons performed TKA procedures at both sites during the study period, with case volumes increasing from approximately 140 TKAs in 1999 to 335 annually in 2018. The proportion of patients referred to the APS also increased over time in accordance with evolving referral criteria.

The APS database is a structured clinical registry in which data are collected at the point of care using predefined fields. Data entry is performed by trained clinical staff as part of routine APS documentation. The structure of the registry and the definitions of recorded variables remained broadly consistent over the study periods, although data capture evolved in parallel with changes in clinical workflows and referral practices. Baseline demographic and perioperative variables were recorded for all patients and included age, sex, type of surgery, anaesthetic technique and regional anaesthesia use. Postoperative variables included analgesic regimens, opioid consumption (expressed as oral morphine equivalents), time to first mobilisation, maximum pain scores at rest and on movement per 24 hours, adverse events (including sedation, pruritus, nausea/vomiting and urinary retention), number of days reviewed by the APS, admission and discharge dates, and use of additional analgesic interventions (e.g., epidural anaesthesia, patient‐controlled analgesia and postoperative regional techniques).

All consecutive adult patients who underwent elective TKA and were referred to the APS were included in the analysis for two distinct time periods: January 1999–December 2004 (Cohort 1) and January 2018–December 2022 (Cohort 2). These intervals were selected due to a statewide restructuring of Orthopaedic services from 2005 until 2017, during which time TKA procedures were performed at other institutions outside of the study sites and APS referral system. Informed consent was obtained from all patients enrolled in the APS database as part of the routine surgical and anaesthetic consent process. Data were stored in a password‐protected database maintained on the hospital’s secure network. Ethics approval for this study was obtained from the Southern Adelaide Local Health Network Human Research Ethics Committee (SALHN/172.22).

### 2.1. Statistical Analysis

Categorical variables were presented as frequencies and percentages and compared using chi‐squared tests. Continuous variables were reported as means with standard deviations. As the continuous variables in the patient demographic section met the assumption of normality based on Kolmogorov–Smirnov testing, unpaired *t*‐tests were used for descriptive group comparisons where appropriate. Baseline demographic characteristics were primarily reported descriptively to characterise the cohorts, whereas inference regarding postoperative outcomes was based on adjusted regression modelling.

For outcome analyses, cohort (1999–2004 vs. 2018–2022) was included as the primary fixed effect. Logistic mixed‐effects regression (logit link) was used for binary outcomes and linear mixed‐effects regression (Gaussian distribution) for continuous outcomes. All models were adjusted for age, sex, primary versus revision surgery, anaesthesia type, use of intrathecal morphine and number of regional blocks. Hospital was included as a random intercept to account for clustering across institutions. Missing data were assessed prior to analysis and were minimal across the variables included in the adjusted models. Data were assumed to be missing at random. Given the low proportion of missingness, a complete‐case analysis was performed, and multiple imputation was not undertaken.

All statistical analyses were performed using STATA Version 18.0 (StataCorp, College Station, Texas, USA). A *p* value of less than 0.05 was considered statistically significant.

## 3. Results

A total of 1273 patients who underwent TKA and were referred to the APS were included in the analysis: 150 patients in Cohort 1 (1999–2004) and 1123 patients in Cohort 2 (2018–2022). Patients in both cohorts were of comparable age. Cohort 2 included a higher proportion of female patients and revision arthroplasties compared with Cohort 1. Baseline demographic characteristics are summarised descriptively in Table 1.

**TABLE 1:** Baseline demographics and perioperative data.

	Cohort 1 (1999–2004) *N* = 150	Cohort 2 (2018–2022) *N* = 1123	*p* value
Age in years, mean (SD)	69.42 (9.3)	69.18 (9.4)	0.77
Gender, *n* (%)			0.01
Female	76 (50.7)	682 (61.3)	
Male	74 (49.3)	430 (38.7)	
Primary vs. Revision			**0.03**
Primary	150 (100)	1087 (96.8)	
Revision	0 (0)	36 (3.2)	
Anaesthesia type, *n* (%)			**< 0.001**
General	113 (75.3)	38 (3.4)	
Spinal	36 (24.0)	1082 (96.4)	
Not recorded	1 (0.7)	3 (0.3)	
Use of intrathecal morphine	8 (5.3)	484 (43.1)	**< 0.001**
Number of regional anaesthesia blocks			**< 0.001**
0	148 (98.7)	289 (25.7)	
1	2 (1.3)	704 (62.7)	
2	0 (0)	125 (11.1)	
3	0 (0)	5 (0.4)	
Regional anaesthesia type^†^			
Adductor canal block	0 (0)	803 (71.5)	**< 0.001**
iPACK block	0 (0)	98 (8.7)	**< 0.001**
Femoral nerve block	0 (0)	17 (1.5)	0.13
Sciatic nerve block	1 (0.7)	18 (1.6)	0.37
Painbuster	0 (0)	17 (1.5)	0.13
Others	1 (0.7)	16 (1.4)	0.45

*Note:* Bold values indicate statistical significance (*p* < 0.05).

Abbreviation: SD = standard deviation.

^†^Patients may receive more than one regional anaesthetic.

**FIGURE 1:**
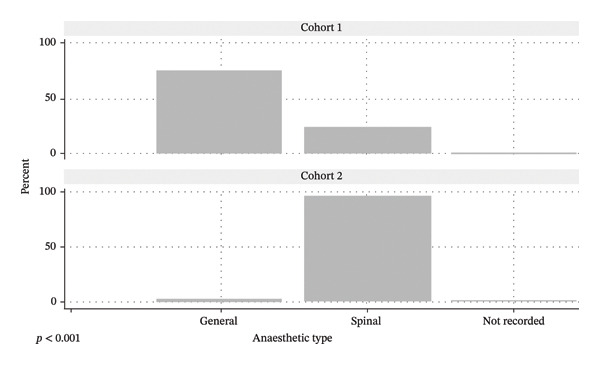
Anaesthetic type by cohort.

**FIGURE 2:**
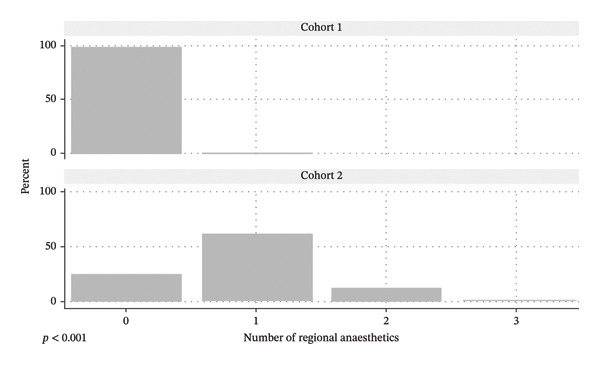
Number of regional anaesthetic techniques.

Intraoperatively, spinal anaesthetic was used significantly more frequently in Cohort 2 compared with Cohort 1 (96.4% versus 24.0%, *p* < 0.001) (Figure 1). The use of regional anaesthesia also increased markedly over the studied time period. Only two patients (1.3%) in Cohort 1 received a regional anaesthetic block, while 969 (74.3%) of the patients in Cohort 2 received at least one regional anaesthetic block. Of this group, 62.7% (*n* = 704) received a single block and 11.5% (*n* = 130) received two or more blocks (Figure 2). The most commonly administered block in Cohort 2 was an adductor canal block (ACB) (71.5%, *n* = 803), followed by the infiltration between popliteal artery and capsule of the knee (iPACK) (8.7%, *n* = 98). Neither of these blocks was performed in Cohort 1 (*p* < 0.001), in which only femoral and sciatic nerve blocks were performed.

Postoperative pain management differed significantly between cohorts. After multivariable adjustment, patients in Cohort 2 had significantly lower odds of requiring invasive pain management strategies (OR: 0.02 and 95% CI: 0.00 to 0.09; *p* < 0.001) (Table 2). Adjusted analyses also demonstrated earlier mobilisation in Cohort 2 (*β*: −0.39 days and 95% CI: −0.69 to−0.10; *p* = 0.009), fewer days of APS review (*β*: −0.59 days and 95% CI −0.72 to−0.45; *p* < 0.001) and a shorter overall length of hospital stay (*β*: −2.44 days and 95% CI: −3.84 to−1.04; *p* = 0.001). Resting pain scores on postoperative day one were not significantly different between cohorts after adjustment. However, movement‐related pain on postoperative day one was modestly higher in Cohort 2 (*β*: 1.00 and 95% CI: 0.12 to 1.88; *p* = 0.02). In addition, Cohort 2 patients had significantly higher postoperative day one opioid requirements (*β*: 55.19 mg oral morphine equivalents and 95% CI: 34.29 to 76.10; *p* < 0.001). Rates of pruritus and postoperative nausea and vomiting did not differ significantly between cohorts following adjustment (Table 2).

**TABLE 2:** Outcome measures.

	Cohort 1 (1999–2004) *N* = 150	Cohort 2 (2018–2022) *N* = 1123	Regression coefficient; 95% confidence interval; *p* value
Invasive pain management strategies, *n* (%)	24 (16.00)	22 (1.96)	OR = 0.02; 0.00–0.09; < 0.001
Epidural	1 (0.67)	0 (0)	
Patient‐controlled anaesthesia	19 (12.67)	14 (1.25)	
Infusion	1 (0.67)	3 (0.27)	
Intrathecal anaesthesia	0 (0)	1 (0.09)	
Regional techniques	3 (2.00)	4 (0.36)	
None	126 (84.00)	1101 (98.04)	
First POD mobilisation, mean (SD)	1.86 (0.54)	1.39 (1.05)	***β* = −0.39; −0.69–−0.10; 0.009**
Resting pain on POD 1, mean (SD)	3.25 (2.27)	3.40 (2.44)	*β* = 0.33; −0.48–1.13; 0.424
Movement pain on POD1, mean (SD)	5.93 (2.54)	6.09 (2.66)	***β* = 1.00; 0.12–1.88; 0.025**
POD 1 opioid requirement in oral morphine equivalent units, mean (SD)	33.90 (56.02)	54.66 (77.79)	***β* = 55.19; 34.29–76.10; < 0.001**
Pruritis POD1	15 (10.00)	61 (5.43)	OR = 0.70; 0.24–2.00; 0.502
Postoperative nausea/vomiting POD1	25 (17.67)	208 (18.52)	OR = 0.74; 0.32–1.71; 0.487
Days reviewed, mean (SD)	1.76 (1.00)	1.08 (0.35)	***β* = −0.59; −0.72–−0.45; < 0.001**
Length of stay, mean (SD)	5.80 (2.69)	3.27 (2.88)	***β* = −2.44; −3.84–−1.04; 0.001**

*Note:* Bold values indicate statistically significant findings (*p* < 0.05).

Abbreviations: POD = postoperative day, SD = standard deviation.

## 4. Discussion

This retrospective historical comparison reviewed trends in anaesthetic practice for TKA across two distinct patient cohorts at a major tertiary hospital network in Australia. The results demonstrate a significant evolution in both neuraxial and regional anaesthesia practices over two decades, reflecting broader international shifts in perioperative care for joint arthroplasty. Importantly, the observed associations in postoperative outcomes persisted after multivariable adjustment for demographic and perioperative factors, with clustering accounted for at the hospital level.

### 4.1. Neuraxial Anaesthesia Trend

One of the most striking findings of this study is the clearly documented transition from general to neuraxial anaesthesia in TKA. The increased adoption of spinal anaesthesia in recent years mirrors a growing body of literature evidence suggesting its potential superiority over general anaesthesia for lower limb arthroplasty, with potential benefits including reduced length of stay and perioperative morbidity [7–11]. Contemporary literature has demonstrated that neuraxial anaesthesia for TKA may be associated with lower risk of venous thromboembolism, blood transfusion, pulmonary complications (including hospital‐acquired pneumonia) and acute renal failure compared with general anaesthesia [9, 12] though the quality of the evidence supporting these claims has generally been characterised as low to moderate within international consensus recommendations [13]. The near‐universal uptake of spinal anaesthesia in Cohort 2 reflects the real‐world translation of this evidence into contemporary practice.

### 4.2. Regional Anaesthesia Development

As greater emphasis has been placed on early postoperative mobilisation following joint replacement, there has been a corresponding rise in the use of motor‐sparing regional anaesthesia techniques. Traditional peripheral nerve blocks, such as femoral and sciatic nerve blocks, while effective for analgesia, have been associated with motor blockade, delay in postoperative mobilisation and an increased falls risk [14]. As a result, novel techniques that preserve motor function, facilitated by advancements in ultrasound technology, have gained popularity.

The ACB, first described in the late 1990s and subsequently popularised as an ultrasound‐guided technique in the 2010s, provides effective analgesia to the medial aspect of the knee while minimising quadriceps motor weakness [15–17]. Similarly, the iPACK technique, first described in 2012, provides targeted posterior knee analgesia through blockade of terminal sensory branches of the sciatic nerve while largely preserving motor function [18, 19]. Contemporary evidence supports the incorporation of ACB into multimodal analgesia pathways for TKA, demonstrating improvements in postoperative pain control, reduced opioid consumption, enhanced early range of motion and improved patient‐reported satisfaction when compared with more proximal motor‐sparing blocks such as the femoral nerve block [20–24]. More recent studies suggest that adding iPACK to ACB may provide additional analgesic benefit in selected patients, particularly with regard to posterior knee pain [25, 26]. However, the evidence supporting routine use of iPACK remains evolving, and international guidelines continue to most consistently recommend multimodal strategies incorporating ACB and local infiltration analgesia (LIA) within enhanced recovery pathways [6, 27–30].

In the present study, regional anaesthesia was rarely used in Cohort 1 but was administered to approximately three‐quarters of patients in Cohort 2, with ACB forming the predominant technique. Uptake of the iPACK block was also notable in the latter cohort. At our institution, the regional anaesthesia techniques evaluated during the study period were predominantly single‐shot peripheral nerve blocks rather than continuous catheter‐based techniques. Despite recommendations supporting LIA as part of enhanced recovery protocols and international guidelines, intraoperative LIA use was not consistently recorded within the historical APS database and, therefore, could not be reliably evaluated in this study. The increased adoption of regional anaesthesia (including ACB and iPACK) in the contemporary cohort likely reflects accumulating evidence, evolving guideline recommendations and improved access to ultrasound‐guided techniques and is conceptually aligned with the goals of modern multimodal, motor‐sparing analgesic strategies that also incorporate LIA.

### 4.3. Postoperative Mobilisation, APS Follow‐Up and Length of Stay

After adjustment for confounders, patients in Cohort 2 mobilised earlier, required fewer days of APS review and had shorter hospital stays. These improvements likely reflect broader multifactorial changes in perioperative care rather than the isolated effect of any single anaesthetic technique. Over the past 2 decades, enhanced recovery and fast‐track arthroplasty pathways have increasingly emphasised early physiotherapy involvement, accelerated mobilisation protocols, standardised multimodal analgesia, reduced inpatient length of stay and coordinated multidisciplinary perioperative care [5]. The evolution in anaesthetic practice observed in the present study should, therefore, be interpreted as one component of a wider institutional and international shift toward recovery‐focused perioperative management for TKA patients.

Although resting pain scores were similar between cohorts, movement‐related pain on postoperative day one was modestly higher in Cohort 2 after adjustment. This difference falls below the minimal clinically important difference (MCID) for pain after TKA, reported as 1.86 on a 0–10 scale [31]. Therefore, the clinical relevance is likely minimal, particularly in the context of concurrent improvements in early mobilisation and shorter hospital stays. It is plausible that the adoption of earlier mobilisation protocols in the contemporary cohort contributed to higher recorded dynamic pain scores without adversely affecting overall recovery trajectories.

### 4.4. Postoperative Opioid Use

After multivariable adjustment, opioid consumption on postoperative day one was significantly higher in the later cohort, suggesting that temporal changes in prescribing practices likely played an independent role beyond differences in the anaesthetic technique or patient characteristics. Indeed, earlier eras of postoperative care were marked by under‐recognition and undertreatment of acute pain [32]. Subsequent guidelines advocated for routine pain assessment, regular documentation of pain intensity scores, early and prompt treatment of pain and patient education [33]. This further evolved to include patients and their families in the development of pain management plans, as well as the concept of multimodal analgesia regimens [34].

At the institutions in this study, pro re nata (PRN) prescribing intervals for opioid administration changed from 4–6 hourly in 2000 to 1‐2 hourly in 2020. Such changes may have contributed to the higher overall prescription and use of opioids in this cohort [35]. Moreover, the higher proportion of general anaesthesia in Cohort 1 may have led to increased intraoperative opioids use and subsequent postoperative sedation, thereby possibly reducing patient demand for analgesia in the early postoperative period.

Importantly, increased opioid consumption was not associated with higher rates of opioid‐related side effects after adjustment. Rates of pruritus, nausea and vomiting were comparable between cohorts, likely reflecting improvements in anticipatory prescribing of antiemetics and bowel regimens, as well as enhanced patient education [35]. In addition, opioid‐sparing strategies, particularly intravenous dexamethasone, have become increasingly incorporated into contemporary multimodal analgesia and enhanced recovery protocols for TKA. Given its recognised analgesic, anti‐inflammatory and antiemetic properties, perioperative dexamethasone use may also have contributed to differences in postoperative recovery between cohorts; however, its administration was not routinely recorded in the APS database.

### 4.5. Limitations

Several limitations warrant consideration. As a retrospective study, analysis was limited to variables routinely collected by the APS at the time of care. Important covariates such as body mass index (BMI), American Society of Anaesthesiologists (ASA) classification, perioperative dexamethasone administration, LIA, tourniquet use, fixation strategy (e.g., cemented versus hybrid techniques), surgical workflow changes and implementation of formal ERAS/Fast‐Track pathways were consistently captured within the historical APS registry and, therefore, could not be incorporated into the adjusted analyses. These variables have now been included in the APS database for future data collection and analysis.

Reliance on the APS database as the primary data source may also limit the completeness of intraoperative anaesthesia data capture, as the registry was originally designed to document postoperative analgesia rather than detailed intraoperative management. Although retrospective data cleaning was performed to extract relevant intraoperative variables, the dataset was not specifically structured for detailed intraoperative analysis. Furthermore, registry input evolved over time in parallel with changes in referral practices, clinical workflows, and perioperative care pathways. In particular, APS referral criteria became more standardised over time, with mandatory referral introduced after 2009 for patients receiving spinal or regional anaesthesia and for procedures associated with significant postoperative pain, including all TKAs. These temporal changes in registry input, referral criteria and case ascertainment may represent a source of systematic bias when comparing historical cohorts.

Although mixed‐effects modelling allowed adjustment for measured confounders and accounted for clustering by hospital, unmeasured confounding remains possible. Additionally, this study does not account for surgical advancements over time, such as the introduction of robotic‐navigated TKA or changes in implant design and technology, which may have independently influenced patient outcomes regardless of the anaesthesia technique. It is also important to recognise that changes in dynamic and static pain scores, as well as length of hospital stay, may have been influenced by evolving institutional practices, recovery protocols and patient expectations, rather than anaesthetic approach alone. Consequently, the associations observed between contemporary anaesthetic practice and postoperative outcomes cannot be fully separated from concurrent changes in rehabilitation pathways, physiotherapy practices, discharge planning and broader enhanced recovery protocols that evolved during the study period.

Finally, this study reflects the experience of a single tertiary hospital network and, therefore, may not be fully generalisable to healthcare settings with differing perioperative pathways, patient case‐mix or anaesthetic practices. Nonetheless, the temporal trends observed closely parallel broader national and international developments in perioperative TKA management reported in the literature, supporting the broader contextual relevance of these findings [6, 9, 12, 36–38].

## 5. Conclusion

This retrospective analysis describes substantial temporal changes in perioperative anaesthetic and analgesic practice for TKA within a single tertiary hospital network over two decades. Contemporary care pathways were associated with earlier mobilisation, reduced reliance on invasive pain management strategies and shorter hospital stays. However, these findings likely reflect broader evolution in institutional perioperative management rather than the isolated effect of any individual anaesthetic technique. While the observed trends align with broader national and international practice patterns, the findings should be interpreted within the context of a single‐centre retrospective study design.

## Author Contributions

Brigid Brown, BMBS: conceived, designed and submitted to Ethics and Governance the relevant protocols. This author also prepared the drafts, analysed and prepared the data, and approved and submitted the final manuscript.

Maxim Nagorny, MD: conceived the study design, assisted with data cleaning and approved the final manuscript.

Joseph Jang, MD: conceived the study design, assisted with data cleaning and approved the final manuscript.

Emily H. Jaarsma, MD: contributed to drafting of the manuscript by writing and editing for clarity in response to reviewer comments.

Tim Soon Cheok, MD: conceived the study design, conducted the statistical analysis, critically revised the drafts and approved the final manuscript.

Charlotte Hall: conceived the study design, assisted with data collection and approved the final manuscript.

Christopher Horwood, PhD: assisted with data linkage and epidemiological study design and approved the final manuscript.

Hidde M. Kroon, MD PhD: assisted with the study design, critically revised the drafts and approved the final manuscript.

Ruurd L. Jaarsma, MD PhD: assisted with the study design, lent departmental support, revised the drafts and approved the final manuscript.

D.‐Yin Lin, MBBS: conceived the study design, critically revised the drafts and approved the final manuscript.

## Funding

The authors have no sources of funding to declare for this manuscript. Open access publishing facilitated by Flinders University, as part of the Wiley ‐ Flinders University agreement via the Council of Australasian University Librarians.

## Ethics Statement

The local Human Research Ethics Committee granted multicentre approval (SALHN/172.22). Informed consent was obtained from all participants.

## Consent

Consent for publication was included in the initial informed consent from all participants. We as an author group also approve this manuscript and give consent for publication.

## Conflicts of Interest

The authors declare no conflicts of interest.

## Data Availability

The data that support the findings of this study are available on reasonable request to the corresponding author and with approval from the Southern Adelaide Local Health Network Human Research Ethics Committee. Data have been de‐identified and stored in a secure institutional database and is not publicly available due to privacy restrictions.
